# Development and validation of a clinical nomogram for predicting suboptimal concentration of valproate in pediatric with epilepsy: a retrospective study

**DOI:** 10.3389/fphar.2026.1811879

**Published:** 2026-04-24

**Authors:** Tianxin Hu, Chunyan Du, Li Lan, Nini Zhang, Yan Zhao, Xun Jiang, Qi Yang, Shanshan Xiao

**Affiliations:** 1 Department of Pharmacy, Tangdu Hospital, The Fourth Military Medical University, Xi’an, Shaanxi, China; 2 Department of Pediatrics, Tangdu Hospital, The Fourth Military Medical University, Xi’an, Shaanxi, China

**Keywords:** Boruta algorithm, epilepsy, nomogram, pediatrics, pharmacokinetics, predictive model, therapeutic drug monitoring (TDM), valproate

## Abstract

**Background:**

Valproate, a first-line anti-seizure medication, has a narrow therapeutic range of 50–100 μg/mL. Many children are prescribed insufficient doses of valproate, resulting in inadequate seizure control or potential toxicity. Currently, no predictive algorithms are available to customize treatment according to the specific needs of children. Our objective was to develop a nomogram that predicts the likelihood of suboptimal valproate concentrations in pediatric patients with epilepsy.

**Methods:**

We conducted a single-center retrospective cohort study of pediatric patients with epilepsy aged 2–18 years who were receiving valproate and had steady-state trough concentrations. The primary outcome was the identification of suboptimal valproate concentrations, defined as levels below 50 μg/mL or above 100 μg/mL. The Boruta algorithm was implemented to identify relevant characteristics from demographic, clinical, and pharmacological variables. Significant predictors identified through this process were incorporated into a multivariable logistic regression model, which was subsequently presented as a nomogram. We assessed the model’s performance regarding discrimination using the area under the curve (AUC) and concordance index (C-index), calibration through a calibration plot and the Hosmer-Lemeshow test, and clinical value via decision curve analysis to guarantee robustness. Bootstrap resampling was performed for internal validation.

**Results:**

Among the 121 included patients,38 (31.4%) patients presented with suboptimal concentrations. The Boruta algorithm and multivariate regression analysis identified four predictors: daily valproate dose (mg/kg/d), acute liver injury (ALI), acute kidney injury (AKI), and the concurrent use of meropenem. The model showed excellent discrimination with an AUC of 0.911 (95% CI 0.849–0.974) and an optimism-corrected C-index of 0.902, alongside good calibration. Decision curves showed a clinical net benefit over a broad probability threshold range (3%–99%). AKI (odds ratio [OR] 16.5), meropenem use (OR 17.39), and ALI (OR 10.86) were significantly associated with suboptimal concentrations.

**Conclusion:**

We developed and internally validated a predictive nomogram that integrates dose, AKI, ALI, and meropenem use to assess the risk of suboptimal concentrations of valproate in pediatric epilepsy. This tool can aid in the early identification of high-risk patients, enabling targeted therapeutic drug monitoring.

## 1 Introduction

Epilepsy is a major neurological disorder that affects around 70 million people worldwide, and children have a disproportionately high prevalence of epilepsy and related health issues (Rincon et al., 2021). Valproate is a broad-spectrum anti-seizure medication used as a first-line treatment for seizure types in pediatric patients because of its proven effectiveness (Shen et al., 2022). However, its application is limited by a narrow therapeutic range of 50–100 mg/L and high variability in drug interactions between individuals. This variability requires careful TDM to improve treatment and reduce toxicity (Zhang et al., 2023).

Although following standard dosing regimens, a significant proportion of pediatric patients (approximately 30%–40%) exhibit suboptimal valproate serum concentrations. These suboptimal concentration are important, as they are linked to poor seizure control, disease relapse risk, toxicity increase and lower quality of life (Lan et al., 2021). Currently, dose adjustments are primarily based on empirical methods. This challenge is particularly pronounced in pediatrics.

The current nomograms for valproate primarily predict single-direction concentration abnormalities, which can be classified as subtherapeutic or beyond therapeutic. Nevertheless, in clinical practice, both conditions require prompt recognition and intervention due to their severe risks: inadequate seizure control in the event of low concentrations and potential toxicity in the event of high concentrations. However, there is still lack of a clinically applicable model that simultaneously calculates the overall risk of concentration deviation. By translating complex pharmacokinetic interactions and clinical risk factors into an intuitive visual format, it enables clinicians to rapidly identify patients at heightened risk of concentration abnormalities without requiring specialized software or advanced pharmacokinetic expertise. This facilitates earlier and more targeted therapeutic drug monitoring, allowing resources to be focused on high-risk individuals.

To fill this gap, we established and verified a multivariable prediction model for suboptimal valproate concentrations, defined as levels outside the 50–100 μg/mL therapeutic range. We created a practical nomogram by incorporating independent risk factors such as demographic, clinical, and pharmacological covariates, providing clinicians a quantitative tool to predict individual patient risk prior to or after treatment beginning. This tool has been designed to aid in the early identification of high-risk patients who may benefit from more intensive therapeutic drug monitoring (TDM) and tailored dose adjustment.

## 2 Methods

### 2.1 Study design and setting

We conducted a retrospective cohort study at the Department of Pediatrics, Tangdu Hospital, Fourth Military Medical University, China. This study focused on patients treated between 1 January 2021, and 31 March 2025. The research population included both the general pediatric ward and the Pediatric Intensive Care Unit (PICU), but excluded Out-patients. We obtained clinical and laboratory data from the Hospital Information System (HIS) and therapeutic drug monitoring (TDM) database.

### 2.2 Participants

Inclusion criteria were: (1) age between 2 and 18 years old; (2) diagnosed with epilepsy according to the classification of the International League Against Epilepsy (ILAE) (Rosenfeld et al., 2021); (3) treated with valproate sodium for a minimum7 days to ensure steady-state pharmacokinetics; (4) measured by a trough serum concentration, that is, a blood sample taken before the next dose; and (5) had good medication compliance (checking nursing records and caregiver reports).

The exclusion criteria were as follows: (1) patients treated with no valproate; (2) those lacking a trough concentration measurement; (3) those who had no steady state of the drug (less than 5 days of consistent dosing before sampling); and (4) those with incomplete medication records that prevented accurate dose or timing calculations.

### 2.3 Outcome and predictors

The main outcome measured was suboptimal valproate serum concentration, categorized as a trough level either below 50 μg/mL or above 100 μg/mL. The standard concentration group, serving as the reference, was defined by trough levels within the effective therapeutic range of 50–100 μg/mL (Yao et al., 2023).

We have recognized that this methodology may integrate particular risk factors influenced by various pathophysiological pathways or drug-drug interaction. However,the limited sample sizes in both the low-concentration and high-concentration groups, there is a risk of overfitting when conducting independent multiple regression analysis (Supplementary Material Section 1 and 2, Page 1, Line 1–69). Therefore, we established a model to simultaneously predict suboptimal factors in clinical demand, conducting comprehensive data evaluation through subgroup-specific descriptive characteristics (Table 1) and subgroup analysis.

**TABLE 1:** Baseline characteristics in pediatric epilepsy patients.

Variables	Total (n = 121)	Min	Max	Suboptimal concentration group				Standard concentration group (n = 83)	*p*
				<50 μg/mL (n = 23)	>100 μg/mL (n = 15)	*p*	Suboptimal concentration group (n = 38)		
Age^a^ (year)	8.4 ± 3.7	2	17	8.3 ± 4.2	10.1 ± 2.7	0.544	9.1 ± 3.6	8.0 ± 3.7	0.154
2–12^b^	92 (76.0)	2	12	16 (69.6)	9 (60)	​	​	67 (80.7)	​
12–18^b^	29 (24.0)	12	17	7 (30.4)	6 (40)	​	​	16 (19.3)	​
Gender^b^	​	​	​	​	​	1	​	​	0.026
Female	28 (23.1)	​	​	3 (13)	1 (6.7)	​	4 (10.5)	24 (28.9)	​
Male	93 (76.9)	​	​	20 (87)	14 (93.3)	​	34 (89.5)	59 (71.1)	​
Weight (kg)^a^	31.4 ± 16.3	8.5	81.2	32.9 ± 21.7	37.1 ± 16.2	0.528	34.6 ± 19.6	29.9 ± 14.4	0.147
2–12 (year)	26.3 ± 11.4	8.5	69	22.3 ± 11.7	30.2 ± 7.0	0.057	​	26.7 ± 11.7	0.625
12–18 (year)	50.9 ± 16.5	25	81.2	58.7 ± 16.3	48.4 ± 19.8	0.321	​	48.4 ± 15.3	0.374
Concentration (μg/mL)^a^	68.5 ± 31.5	1.7	198.2	26.91 ± 17.93	125.9 ± 23.45	<0.001	66.0 ± 52.9	69.7 ± 13.6	0.554
2–12^c^ (year)	65.1 (52.7, 83.3)	1.7	144.5	31.0 (13.9, 40.9)	118.4 (107.5, 127.0)	<0.001	​	66.6 (59.3, 77.5)	0.097
12–18^c^ (year)	70.2 (50.6, 87.2)	2.7	198.2	18.1 (4.4, 40.8)	117.7 (115.2, 132.2)	0.003	​	71.0 (57.9, 79.4)	0.726
Dosage form^b^	​	1	3	​	​	​	​	​	0.034
Sustained release tablet	57 (47.1)	​	​	9 (39.1)	12 (80)	<0.001	21 (55.3)	36 (43.4)	​
Oral solution	62 (51.2)	​	​	14 (60.9)	1 (6.7)	​	15 (39.5)	47 (56.6)	​
Injection	2 (1.7)	​	​	0 (0)	2 (13.3)	​	2 (5.3)	0 (0)	​
Daily dose (g)^c^	0.5 (0.4, 0.8)	0.15	5	0.5 (0.4, 0.6)	1.0 (0.9, 2.0)	<0.001	0.6 (0.5, 1.0)	0.5 (0.4, 0.7)	0.015
2–12^a^ (year)	0.6 ± 0.3	0.15	2	0.5 ± 0.1	1.1 ± 0.5	<0.001	​	0.5 ± 0.2	0.004
12–18^c^ (year)	1.0 (0.8, 2.0)	0.25	5	1.0 (0.6, 1.5)	2.0 (1.2, 2.0)	0.101	​	0.9 (0.8, 1.0)	0.149
Daily dose (mg/kg/d)^a^	23.2 ± 13.3	5	118.48	22.1 ± 9.5	38.9 ± 25.3	0.006	28.8 ± 19.1	20.7 ± 8.7	0.002
2–12^a^ (year)	22.6 ± 10.3	5	54.55	23.9 ± 8.9	34.8 ± 14.2	0.019	​	20.4 ± 8.6	<0.001
12–18^c^ (year)	25.6 (13.2, 29.4)	7.25	118.48	15.4 (11.7, 23.5)	31.9 (28.2, 49.2)	0.022	​	18.9 (13.0, 30.4)	0.538
Hemoglobin (g/L)^a^	121.1 ± 13.1	82	151	115.4 ± 20.4	122.3 ± 11.8	0.242	118.1 ± 17.7	122.5 ± 10.1	0.088
2–12 (year)	119.1 ± 12.4	82	143	108.4 ± 18.4	122.5 ± 11.9	0.032	​	121.1 ± 9.3	0.009
12–18 (year)	130.8 ± 11.1	103	151	132.9 ± 11.6	126.0 ± 12.5	0.327	​	131.6 ± 10.5	0.648
WBC×10^9/^L^a^	7.2 ± 2.7	3.12	21.77	7.9 ± 4.1	7.0 ± 3.0	0.474	7.5 ± 3.7	7.1 ± 2.0	0.381
2–12 (year)	7.5 ± 2.8	3.94	21.77	8.7 ± 4.4	7.2 ± 3.6	0.339	​	7.2 ± 2.0	0.166
12–18 (year)	6.1 ± 1.7	3.12	10.76	5.3 ± 2.0	6.5 ± 1.6	0.264	​	6.2 ± 1.7	0.570
Albumin (g/L)^a^	40.9 ± 6.9	28.8	80	39.9 ± 10.5	38.8 ± 4.3	0.693	39.5 ± 8.6	41.6 ± 6.0	0.125
2–12 (year)	40.4 ± 6.3	28.8	80	37.9 ± 6.1	37.6 ± 4.2	0.866	​	41.4 ± 6.3	0.008
12–18 (year)	42.9 ± 8.1	30	80	44.9 ± 16.4	41.4 ± 2.4	0.614	​	42.6 ± 3.0	0.819
ALI^b^	​	​	​	​	​	<0.001	​	​	<0.001
No	107 (88.4)	​	​	22 (95.7)	5 (33.3)	​	27 (71.1)	80 (96.4)	​
Yes	14 (11.6)	​	​	1 (4.3)	10 (66.7)	​	11 (28.9)	3 (3.6)	​
AKI^b^	​	​	​	​	​	0.012	​	​	<0.001
No	108 (89.3)	​	​	20 (87)	7 (46.7)	​	27 (71.1)	81 (97.6)	​
Yes	13 (10.7)	​	​	3 (13)	8 (53.3)	​	11 (28.9)	2 (2.4)	​
Meropenem^b^	​	​	​	​	​	<0.001	​	​	<0.001
No	103 (85.1)	​	​	9 (39.1)	14 (93.3)	​	23 (60.5)	80 (96.4)	​
Yes	18 (14.9)	​	​	14 (60.9)	1 (6.7)	​	15 (39.5)	3 (3.6)	​
Lacosamide^b^	​	​	​	​	​	0.436	​	​	0.017
No	106 (87.6)	​	​	19 (82.6)	10 (66.7)	​	29 (76.3)	77 (92.8)	​
Yes	15 (12.4)	​	​	4 (17.4)	5 (33.3)	​	9 (23.7)	6 (7.2)	​
Levetiracetam^b^	​	​	​	​	​	0.63	​	​	0.213
No	97 (80.2)	​	​	19 (82.6)	14 (93.3)	​	33 (86.8)	64 (77.1)	​
Yes	24 (19.8)	​	​	4 (17.4)	1 (6.7)	​	5 (13.2)	19 (22.9)	​
Oxcarbazepine^b^	​	​	​	​	​	0.395	​	​	0.431
No	114 (94.2)	​	​	23 (100)	14 (93.3)	​	37 (97.4)	77 (92.8)	​
Yes	7 (5.8)	​	​	0 (0)	1 (6.7)	​	1 (2.6)	6 (7.2)	​
Lamotrigine^b^	​	​	​	​	​	0.223	​	​	0.82
No	97 (80.2)	​	​	20 (87)	10 (66.7)	​	30 (78.9)	67 (80.7)	​
Yes	24 (19.8)	​	​	3 (13)	5 (33.3)	​	8 (21.1)	16 (19.3)	​
Clonazepam^b^	​	​	​	​	​	0.259	​	​	0.286
No	81 (66.9)	​	​	15 (65.2)	13 (86.7)	​	28 (73.7)	53 (63.9)	​
Yes	40 (33.1)	​	​	8 (34.8)	2 (13.3)	​	10 (26.3)	30 (36.1)	​
Perampanel^b^	​	​	​	​	​	1	​	​	0.229
No	101 (83.5)	​	​	20 (87)	14 (93.3)	​	34 (89.5)	67 (80.7)	​
Yes	20 (16.5)	​	​	3 (13)	1 (6.7)	​	4 (10.5)	16 (19.3)	​
Topiramate^b^	​	​	​	​	​	0.63	​	​	0.318
No	110 (90.9)	​	​	19 (82.6)	14 (93.3)	​	33 (86.8)	77 (92.8)	​
Yes	11 (9.1)	​	​	4 (17.4)	1 (6.7)	​	5 (13.2)	6 (7.2)	​
Vigabatrin^b^	​	​	​	​	​	​	​	​	0.324
No	116 (95.9)	​	​	23 (100)	15 (100)	1	38 (100)	78 (94)	​
Yes	5 (4.1)	​	​	0 (0)	0 (0)	​	0 (0)	5 (6)	​
Zonisamide^b^	​	​	​	​	​	0.509	​	​	0.589
No	117 (96.7)	​	​	21 (91.3)	15 (100)	​	36 (94.7)	81 (97.6)	​
Yes	4 (3.3)	​	​	2 (8.7)	0 (0)	​	2 (5.3)	2 (2.4)	​
Phenobarbital^b^	​	​	​	​	​	0.149	​	​	0.097
No	119 (98.3)	​	​	23 (100)	13 (86.7)	​	36 (94.7)	83 (100)	​
Yes	2 (1.7)	​	​	0 (0)	2 (13.3)	​	2 (5.3)	0 (0)	​

^a^ Mean ± SD.

^b^ n (%).

^c^ IQR, interquartile range; WBC, white blood cell; ALI, acute liver injury; AKI, acute kidney injury.

Candidate predictors were selected based on clinical relevance and the literature (Yao et al., 2023). These predictors included demographic and clinical variables such as age (years), sex, and body weight (kg). Pharmacological variables considered were the valproate daily dose (mg/kg/d), dosage form (tablet, oral solution, or injection) and concurrent use of other anti-seizure medication (e.g., lacosamide, levetiracetam) or antimicrobials (e.g., meropenem) known or suspected to interact with valproate metabolism was also recorded. The laboratory variables included hemoglobin (g/L), white blood cell count (WBC×10^9^/L), and serum albumin (g/L). Additionally, the presence of acute liver injury (ALI) or acute kidney injury (AKI) at the time of TDM was recorded.

### 2.4 Data collection and concentration measurement

Data were extracted from the HIS by trained pharmacists, and any discrepancies were resolved by a pediatric neurologist. Administer the initial dose according to the drug package insert and collected 3 mL of blood sample after 7 days of unchanged therapy. For patients requiring dose adjustment based on measured results, physicians should implement individualized dose adjustment protocols and then collect blood samples after 7 days of treatment. The samples were centrifuged at 3,500 rpm for 5 min. Serum valproate concentration was measured using a Siemens valproate assay kit on a Siemens Viva-ProE fully automated biochemical analyzer, according to the manufacturer’s instructions. The coefficient of variation was less than 5% for internal quality control.

This study encompassed three formulations of valproate: oral solution, sustained-release tablets, and injectable form. The medicine package insert indicates that the injection has a bioavailability of 100%, the oral solution demonstrates bioavailability around 100% (Sanofi-Aventis, 2020a), and the sustained-release tablets are bioequivalent to the immediate-release formulation, with an overall bioavailability of roughly 100% (Sanofi-Aventis, 2020b; Perucca, 2002). We determined that the three dose formulations exhibited bioequivalence. As a result, steady-state trough concentrations were similar across various dose formulations (Patsalos et al., 2008).

### 2.5 Statistical analysis

Statistical analyses were conducted using R software version 3.3.2 (http://www.R-project.org, The R Foundation) and Free Statistics software (version 2.4.0) (Ma et al., 2023). A two-sided *P*-value of less than 0.05 was considered statistically significant. The analysis followed the TRIPOD guidelines for the model development.

We compared the baseline characteristics between the suboptimal and standard concentration groups. Categorical variables are presented as frequencies and percentages and were compared using chi-squared test or Fisher exact test, as appropriate. The normality of continuous variables was assessed using the Shapiro- Wilk test, which is particularly sensitive and reliable for small sample sizes. Normal distribution variables are mean and standard deviation, comparisons are performed with Student’s t-test, non-normally distributed variables are median and interquartile range, and comparisons are performed with the Mann-Whitney U test.

To select the most relevant predictors for our multivariable model, we used the Boruta algorithm, a random forest-based wrapper method designed to identify all variables that are relevant to the outcome (Yan et al., 2024). The algorithm iteratively compares the importance of each original variable with that of shadow variables (randomly permuted copies). Variables with significantly higher importance than the shadow variables are classified as “confirmed important,” while those with lower importance are classified as “rejected.” Only the confirmed important variables were retained for subsequent multivariable logistic regression analysis comparing suboptimal vs. standard concentrations.

The final logistic regression model was converted into a nomogram using the R package rms, and the individual risk was assessed. The model performance was measured using discrimination and calibration. Discrimination, which measures the model’s ability to distinguish between patients with and without the outcome, was assessed using the area under the receiver operating characteristic curve (AUC) and concordance index (C-index), both accompanied by 95% confidence intervals (CI). The calibration measure of agreement between the predicted probabilities and observed results was visualized using a calibration plot and statistically tested using the Hosmer-Lemeshow goodness-of-fit test. To avoid over-optimism of the development data, all performance measures (AUC, C-index, and calibration slope) were internally tested with 1,000 bootstrap resamples. Additionally, a leave-one-out cross-validation was conducted to further validate the model. The potential net clinical benefit of using the prediction model across different probability levels was quantified using decision curve analysis (Bo et al., 2024).

The Ethics Committee of Tangdu Hospital, Fourth Military Medical University, approved this study (Approval No. K-HG-202601-11). Since this was a retrospective and observational study that used anonymized data, the Institutional Review Board did not require individual informed consent. All procedures adhered to the Declaration of Helsinki.

## 3 Results

### 3.1 Baseline characteristics in pediatric epilepsy patients

**FIGURE 1:**
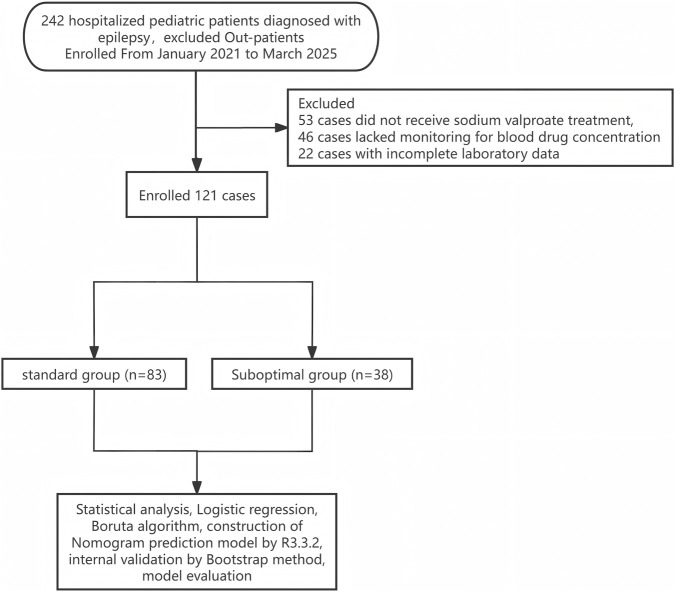
Flow diagram of study population enrollment.

Between January 2021 and March 2025, 242 children were diagnosed with epilepsy at the pediatric ward of Tangdu Hospital, Fourth Military Medical University. After excluding 53 cases without valproate treatment, 46 cases without serum drug concentration monitoring, and 22 cases with incomplete laboratory data, 121 pediatric patients who received valproate therapy were included in the final study (Figure 1). Based on steady-state trough serum concentrations, patients were categorized into two groups: 38 patients (31.40%) in the suboptimal concentration group (valproate concentration <50 μg/mL or >100 μg/mL) and 83 patients (68.60%) in the standard concentration group (valproate concentration 50–100 μg/mL). The suboptimal concentration group was further divided: 23 patients (19.01%) had concentrations <50 μg/mL, and 15 patients (12.40%) had concentrations >100 μg/mL. The baseline characteristics of the cohort are presented in Table 1. The mean age was 8.4 ± 3.7 years, with no significant age difference between the suboptimal and standard groups (9.1 ± 3.6 vs. 8.0 ± 3.7 years, *P* = 0.154). Sex distribution, body weight, and key laboratory parameters (hemoglobin, WBC, and albumin) were similar across the groups (all P > 0.05). However, significant differences were noted in terms of clinical complications and concomitant medications. The suboptimal concentration group had a higher prevalence of AKI (28.9% vs. 2.4%, P < 0.001), ALI (28.9% vs. 3.6%, P < 0.001), and meropenem use (39.5% vs. 3.6%, *P* < 0.001). Specifically, the incidence of AKI reached 53.3% in the >100 μg/mL subgroup, and the incidence of ALI was 66.7% in the same subgroup (*P* < 0.001 for both). Meropenem use was notably high in the <50 μg/mL subgroup (60.9%) and only one case in the >100 μg/mL subgroup (6.7%,*P* < 0.001).

### 3.2 Identify the potential prognostic factors by Boruta algorithm

**FIGURE 2:**
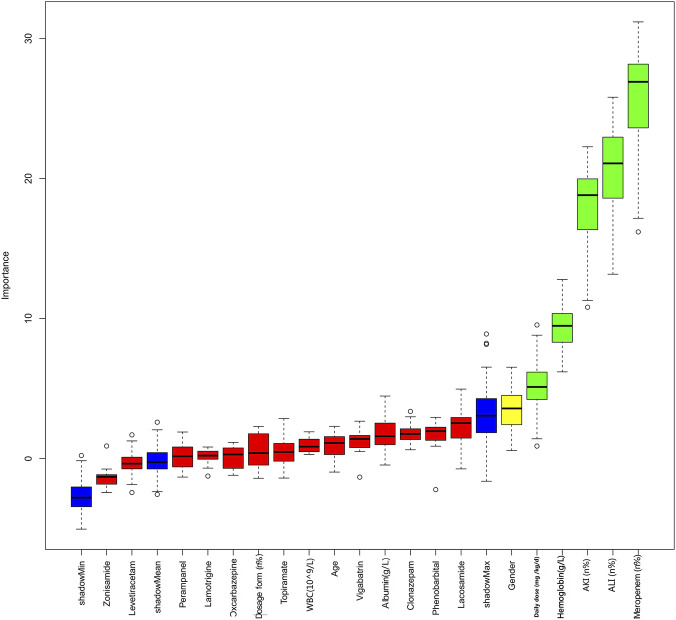
Feature selection based on the Boruta algorithm. The horizontal axis represents the name of each variable, while the vertical axis denotes the Z value of each variable. The box plot illustrates the Z value of each variable throughout the model computation. Green boxes: confirmed important variables. Red boxes: rejected unimportant variables. Blue boxes: tentative variables. Yellow boxes: shadow variables (randomly permuted copies) used as a reference threshold. ShadowMin, ShadowMean, and ShadowMax denote the minimum, mean, and maximum importance values among these shadow variables, respectively.

To select variables for multivariable modeling, we used the Boruta wrapper method based on a random forest classifier. Boruta classifies variables as confirmed important (green), tentative (yellow), and rejected (red). The most important variables (green) were daily dose (mg/kg/d), hemoglobin level, presence of ALI, presence of AKI, and concurrent use of meropenem (Figure 2).

### 3.3 Multivariate regression analysis

The Boruta feature selection identified five variables that were subsequently included in the multivariate logistic regression analysis (Table 2). According to the contributing factors obtained from subgroup analysis (Supplementary Table S1). A dosage increase of 1 daily dose (mg/kg/d) was associated with an estimated 6% elevated risk of supratherapeutic concentration (OR 1.06, 95% CI: 1.02–1.1, *P* = 0.006) in suboptimal model. Patients with ALI faced a nearly 9.86-fold greater risk of supratherapeutic concentrations than those without ALI (OR 10.86, 95% CI: 2.82–41.87, *P* = 0.001). Similarly, patients with AKI exhibited an approximately 15.5-fold increased risk compared to non-AKI patients (OR 16.5, 95% CI: 3.44–79.18, *P* < 0.001). Conversely, According to the contributing factors obtained from subgroup analysis (Supplementary Table S2) each 1 g/L increase in hemoglobin was associated with an approximately 3% reduced risk of subtherapeutic concentration, although this association lacked statistical significance (OR 0.97, 95% CI: 0.95–1, *P* = 0.092) in suboptimal model. Furthermore, patients receiving meropenem had an approximately 16.39-fold higher risk of subtherapeutic concentrations than those not receiving the drug (OR 17.39, 95% CI: 4.63∼65.33, *P* < 0.001) in suboptimal model.

**TABLE 2:** Multivariate regression analysis.

Variable	OR (95% CI)	*P* value
Daily dose (mg/kg/d)	1.06 (1.02∼1.1)	0.006
Hemoglobin	0.97 (0.95∼1)	0.092
ALI	​	​
No	1(Ref)	​
Yes	10.86 (2.82∼41.87)	0.001
AKI	​	​
NO	1(Ref)	​
Yes	16.5 (3.44∼79.18)	<0.001
Meropenem	​	​
No	1(Ref)	​
Yes	17.39 (4.63∼65.33)	<0.001

OR, odds ratios; ALI, acute liver injury; AKI, acute kidney injury.

### 3.4 Comparative analysis of effect sizes across subgroup and optimal group

A systematic comparison of the effect directions for the four predictors across the two subgroups, which compared low vs. optimal and high vs. optimal concentrations groups, revealed that all variables demonstrated identical effect directions, with odds ratios consistently greater than 1 and no evidence of opposing effects (Supplementary Table S3).

### 3.5 Establishment and validation of the nomogram prediction model

**FIGURE 3:**
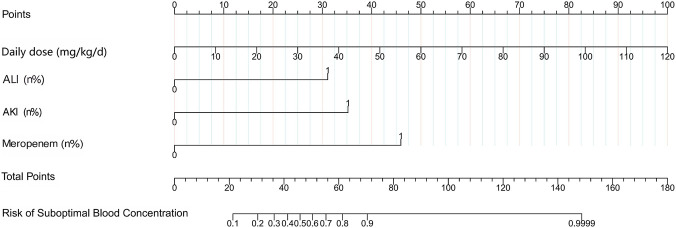
To use the nomogram, locate the patient’s daily valproate dose (mg/kg/d) on the corresponding axis, draw a vertical line upward to the “Points” axis to obtain the score for this variable. Repeat this process for acute liver injury (ALI), acute kidney injury (AKI), and concomitant use of meropenem. Sum the individual scores to obtain the total points, then draw a vertical line downward from the total points axis to the “Risk”axis to determine the predicted probability of suboptimal concentration. (Refer to the Supplementary Material Section 4 for specific User Guide).

The Boruta algorithm and multivariate regression analysis identified four major predictors: daily dose (mg/kg/d), ALI, AKI, and meropenem. These factors were integrated into a multivariable logistic regression model, which was illustrated using a nomogram (Figure 3). The nomogram assigns weighted points to each predictor level, and the sum of the points indicates the predicted probability of a suboptimal valproate concentration.

**FIGURE 4:**
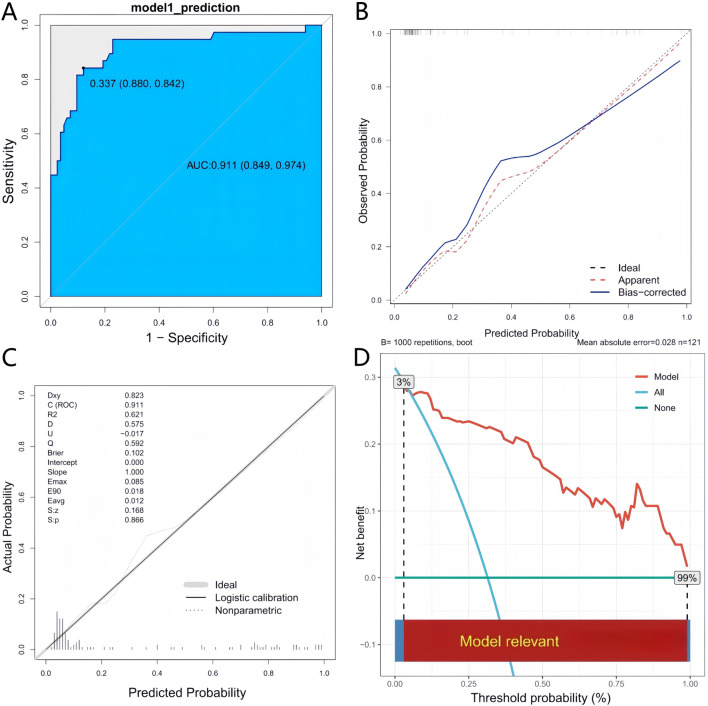
The discrimination and calibration assessment of the model **(A)** ROC curve and AUC of the nomogram within the cohort. **(B)** Calibration curve for the nomogram predicting the likelihood of suboptimal blood concentration, validated using bootstrap sampling. **(C)** Calibration curve for the nomogram predicting the likelihood of suboptimal blood concentration with leave-one-out cross-validation. **(D)** Decision curve for the predictive nomogram. The net benefits were assessed at various threshold probabilities. The red line denotes the prediction nomogram. The blue line denotes the premise that all patients possess poor blood concentration levels. The horizontal line signifies the presumption that no patients experience suboptimal blood concentration.

The model had exceptional discriminative capacity, achieving an AUC of 0.911 (95% CI: 0.849–0.974). The concordance index (C-index) was 0.91. To determine the optimal clinical operating point, we calculated the Youden index (1.7216) and computed the optimal predictive probability cut-off of 0.337. This means that if the predicted probability of suboptimal valproate concentration as ≥ 0.337 (33.7%), the patient is classified as “sub-standard”; otherwise, the patient is classified as “up to standard. At this threshold, the model achieved a sensitivity of 0.842 and a specificity of 0.879 (Figure 4A). Internal validation using 1,000 bootstrap samples resulted in an optimistic corrected C-index of 0.902,underscoring the model’s robust performance. The calibration analysis demonstrated a robust concordance between the predicted probabilities and actual outcomes, as evidenced by the Hosmer-Lemeshow test (χ^2^ = 9.354, *P* = 0.313). The calibration plot exhibited a slope of 0.828, which roughly aligned with the optimal value of 1.0 (Figure 4B). Subsequent validation via 10-fold cross-validation produced an optimism-adjusted C-index of 0.898. The resultant slope of 1.0 and the smooth calibration curve closely adhered to the ideal line, as depicted in the validation calibration curve (Figure 4C). Decision curve analyses were used to assess the clinical net benefit of applying the prediction model to various threshold probabilities. We showed that using the nomogram to guide clinical decisions gives a positive net benefit compared to using the strategy of “treating all” or “treating none” for threshold probabilities between 3% and 99% (Figure 4D).

## 4 Discussion

This study presents the development and internal validation of the first pediatric-specific nomogram for predicting suboptimal valproate serum concentrations below or above the standard dose. The model integrates four clinical variables daily dose (mg/kg/d), ALI, AKI and the concurrent use of meropenem with strong discrimination power with 0.911 AUC and good calibration. A significant innovation is Boruta algorithm for robust feature selection. Unlike regression methods, Boruta identifies a simplified set of predictors for pediatric pharmacokinetic studies.

Based on bioequivalence assumptions, this study examined oral solutions, sustained-release tablets, and injectables. Oral solutions had rapid absorption with peak attainment time of 1–2 h, while sustained-release tablets had slower absorption with peak attainment time of 4–8 h (Perucca, 2002). This study measured steady-state trough concentrations when the drug was in the elimination phase, reflecting exposure levels rather than absorption rates. Differences in absorption rates exhibited little effect on steady-state trough concentrations, which supports the worldwide TDM guidelines’ recognition of comparability across dose forms (Patsalos et al., 2008).

The multivariable regression analysis identified AKI (OR = 16.5), ALI (OR = 10.86), and concomitant meropenem use (OR = 17.39) as the strongest predictors of suboptimal valproate concentrations, each increasing risk by more than ten-fold and underscoring that critically ill patients with organ dysfunction or complex infections are particularly vulnerable. The effect sizes of these pathophysiological factors and drug interactions significantly surpassed that of the daily dose, indicating that dose adjustment alone within the dosage range specified in the package insert is inadequate to reduce risk; therefore, early therapeutic drug monitoring (TDM) and proactive strategies—such as reevaluating antimicrobial selections or starting a loading dose—are necessary. The clinical ramifications of concentration deviations vary by direction: subtherapeutic levels (<50 μg/mL) elevate the risk of breakthrough seizures and status epilepticus, while supratherapeutic levels (>100 μg/mL) correlate with dose-dependent toxicity, encompassing sedation, ataxia, tremor, gastrointestinal disturbances, and, less frequently, hepatotoxicity or hyperammonemia. These specific outcomes underscore the necessity of identifying patients at risk for inadequate concentrations and ascertaining the direction of deviation to inform suitable therapeutic strategies. Therefore, we performed a subgroup analysis on the suboptimal group to ascertain predictive factors (Supplementary Material Section 1 and 2, Page 1, Line 1–69). Meropenem was a major influencing factor in the low-concentration group, while AKI and ALI were significant influencing variables in the high-concentration group. Despite the presence of additional risk factors in the subgroup, they were excluded from the modeling of the suboptimal group due to an insufficient effect size significance.

We compared the direction and magnitude of four core variables across three multivariate models (Supplementary Table S3). Results indicated that all variables exhibited OR values greater than 1 across all models, without any opposite orientations. In particular, meropenem exhibited the most significant effect in the low-concentration model (OR = 41.48,*P* < 0.001) and no significant association in the high-concentration model (OR = 1.90,*P* = 0.588). This is consistent with the mechanism of meropenem, which is to induce sodium valproate metabolism and specifically reduce plasma concentrations. AKI and ALI exhibited exceptionally strong effects in the high-concentration model (OR = 46.29 and 53.33,*P* < 0.001), but weak effects in the low-concentration model (OR = 6.07 and 1.21,*P* = 0.057and *P* = 0.87). These findings are consistent with the mechanisms of decreased drug clearance due to organ dysfunction and concentration elevation caused by accumulation. The high-concentration model exhibited stronger effects on the daily dose (mg/kg/day) (OR = 1.10,*P* < 0.001), whereas the low-concentration model did not exhibit any significant effect (OR = 1.02, *P* = 0.495). That means in the absence of other factors (AKI, ALI, meropenem), dosage alone is insufficient to reliably predict the risk of low concentrations. Therefore, we adopted the minimum dose specified in the drug package insert (20 mg/kg/d) as the applicable minimum dose condition for using this predictive model. In conclusion, suboptimal group offers a comprehensive risk assessment that extends beyond the therapeutic window, while the subgroup exposes mechanism -specificity.The suboptimal model enhanced statistical power, enabling more robust estimation of overall nomogram effects.

Liver dysfunction is a major contributor to elevated valproate concentrations, particularly in pediatric patients whose hepatic enzyme systems are still maturing. Valproate is primarily metabolized in the liver through glucuronidation (mediated by UDP-glucuronosyltransferases, UGT) and β-oxidation, with the former pathway being particularly susceptible to developmental regulation (Mei et al., 2021). In children, the activity of UGT isoforms and other drug-metabolizing enzymes undergoes age-dependent changes, with full maturation often not achieved until adolescence (Yanni, 2012). When liver function is impaired—whether due to underlying disease, drug-induced injury, or developmental immaturity—the activity of these hepatic enzymes decreases, and hepatic blood flow may be reduced. This significantly slows drug metabolism and prolongs the elimination half-life, increasing the risk of drug accumulation and toxicity (Liu et al., 2021). Our subgroup analysis revealed that the prevalence of liver injury was 66.7% in the high-concentration subgroup (>100 μg/mL), compared with only 4.3% in the low-concentration subgroup (<50 μg/mL). This striking disparity suggests that even standard or reduced doses may lead to supratherapeutic concentrations in children with compromised hepatic function (Benic et al., 2021). Given these considerations, clinicians should adopt a cautious approach when initiating or adjusting valproate therapy in pediatric patients with suspected or confirmed liver dysfunction. We recommend starting with a reduced initial dose, considering longer dosing intervals, and implementing early and frequent therapeutic drug monitoring to ensure concentrations remain within the safe and effective range. Future studies incorporating pharmacogenetic markers (e.g., UGT and CYP2C9 polymorphisms) and age-specific metabolic models may further refine dosing strategies in this vulnerable population.

Renal impairment significantly affects drug metabolism. The results showed that renal injury was 53.3% in the high-concentration subgroup compared with 13% in the low-concentration subgroup. Although less than 3% of valproate is excreted unchanged through the kidneys, its metabolites are primarily eliminated via the renal pathways (Ji et al., 2021). In severe renal impairment, these metabolites can accumulate and alter the metabolism of the parent compound via feedback or competitive inhibition (Banawalikar et al., 2021). Valproate is highly protein-bound (approximately 90%), primarily to albumin. In critically ill pediatric patients—particularly those with hypoalbuminemia, renal impairment, or uremia—the free (pharmacologically active) fraction may increase substantially due to reduced protein binding or competitive displacement by endogenous substances. Consequently, a total concentration within or even below the therapeutic range may coexist with a free concentration that is elevated, potentially leading to toxicity without appropriate recognition (Avachat et al., 2022). Therefore, the TDM guidelines of the International League Against Epilepsy (ILAE) explicitly state that monitoring free plasma concentrations rather than total concentrations is recommended in patients with hypoalbuminemia, renal insufficiency, and specific pediatric populations (Patsalos et al., 2008). However, conventional therapeutic drug monitoring and the standard treatment range of 50–100 μg/mL are both based on total plasma concentrations, and we must acknowledge the limitations of this approach.

The notable correlation between concurrent meropenem administration and suboptimal valproate levels highlights the important pharmacokinetic interactions, as meropenem, a broad-spectrum carbapenem antibiotic, induces valproate metabolism (Wu et al., 2023). This induction is mainly attributed to increased glucuronidation of valproate through UGT enzymes and potentially the suppression of renal tubular reabsorption of valproate-glucuronide (VPA-g). These mechanisms lead to a significant enhancement in total body clearance and a swift, dramatic decline in serum valproate levels, frequently diminishing by 60%–90% (Cattaneo et al., 2022). Physicians should consider other antibiotics when possible for severe infections. If meropenem is required, it is advisable to increase the prophylactic empirical dose and monitor serum concentrations for breakthrough seizures.

## 5 Limitations

This study was a single-center retrospective observational analysis, and the potential for unidentified confounding variables may persist despite adjustments for known confounders. For instance, while acute kidney injury (AKI) demonstrated a significant correlation with inadequate serum medication concentrations (*P* < 0.001, OR>1), the precision of the effect size estimate was compromised by the limited sample size of merely 13 patients in this cohort (95%CI:3.44–79.18). The extent of risk escalation (whether suboptimal concentration levels surpassed 1-fold or multiple-fold) remains ambiguous. Comparable imprecision was noted in the ALI cohort and the meropenem combination therapy cohort. Additionally, all possible pathophysiology factors such as detailed liver and kidney function indices, metabolic enzyme genotypes, compliance, absorption disorders and genetic polymorphisms were all not considered.

Another limitation of this study is the lack of external validation. While we employed rigorous internal validation methods—including bootstrap resampling and 10-fold cross-validation—to assess model stability and optimism, these techniques cannot fully account for differences in patient populations, clinical practices, or measurement methods across institutions. External validation, ideally in an independent, prospective, and multi-center cohort, is essential to evaluate the generalizability of our nomogram before it can be confidently applied in routine clinical practice. Without such validation, the model may exhibit optimism bias, meaning its predictive performance could be overestimated, and the estimated effect sizes may not be reproducible in other settings. Therefore, our findings should be considered exploratory. Future efforts should prioritize external validation in larger, diverse pediatric populations to confirm the robustness and clinical utility of the proposed nomogram.

## 6 Conclusion and future directions

In conclusion,by incorporating four accessible clinical predictors—daily dosage,AKI,ALI,and meropenem administration—our nomogram offers a practical and bedside-ready evaluation tool. The found predictors correspond with recognized pharmacokinetic principles. In contrast to earlier pediatric therapeutic drug monitoring (TDM) studies, which primarily employed descriptive methods or concentrated on population pharmacokinetic (PopPK) modeling (Tauzin et al., 2018). This suboptimal model is intended as a screening instrument to provide a supplementary strategy for early risk stratification in high-risk pediatric patients and to intensified therapeutic drug monitoring (TDM),the dosage modification procedure must comply with population pharmacokinetic models and Bayesian predictive techniques. The model produced a positive net benefit due to its robust performance, remarkable discriminative ability, and ability to guide clinical decisions. Future work should focus on prospective external validation through multi-center studies with different populations. Further, adding pharmacogenetic data (CYP2C9 and UGT genotypes) into a larger model may be useful, and would lead to fully personalized anti-seizure medication doses in children.

## Data Availability

The original contributions presented in the study are included in the article/Supplementary Material, further inquiries can be directed to the corresponding authors.
