# Supplementary material for: Development and validation of a clinical nomogram for predicting suboptimal concentration of valproate in pediatric with epilepsy: a retrospective study
Source: Front Pharmacol. 2026 Apr 24;17:1811879. doi: 10.3389/fphar.2026.1811879 (PMC13153454; doi:10.3389/fphar.2026.1811879)
Supplement: Supplementary file 1 [file Supplementaryfile1.docx]

***Supplementary material:***

**1、Establishment and validation of the nomogram prediction model (High vs. Therapeutic)**

Boruta algorithm identify the potential prognostic factors were daily dose (mg/kg/d),AKI, ALI, dosage form, lacosamide, phenobarbital, weight (Supplementary Figure 1). Multivariate analysis further identified the following variables, daily dose(mg/kg/d), AKI, ALI, lacosamide(Supplementary Table 1).These factors were integrated into a multivariable logistic regression model, which was illustrated using a nomogram(Supplementary Figure 2).The nomogram prediction model exhibits overfitting and unstable due to insufficient outcome events (n=15) (Supplementary Figure 3).


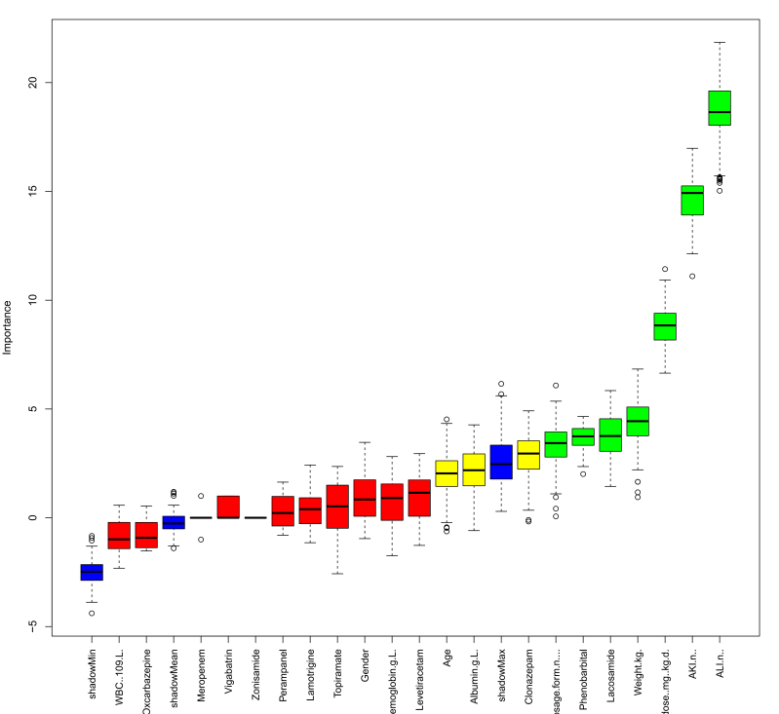


**Supplementary Figure 1** Feature selection based on the Boruta algorithm. The horizontal axis represents the name of each variable, while the vertical axis denotes the Z value of each variable. The box plot illustrates the Z value of each variable throughout the model computation. The green boxes denote significant variables, whereas the red boxes indicate insignificant variables.


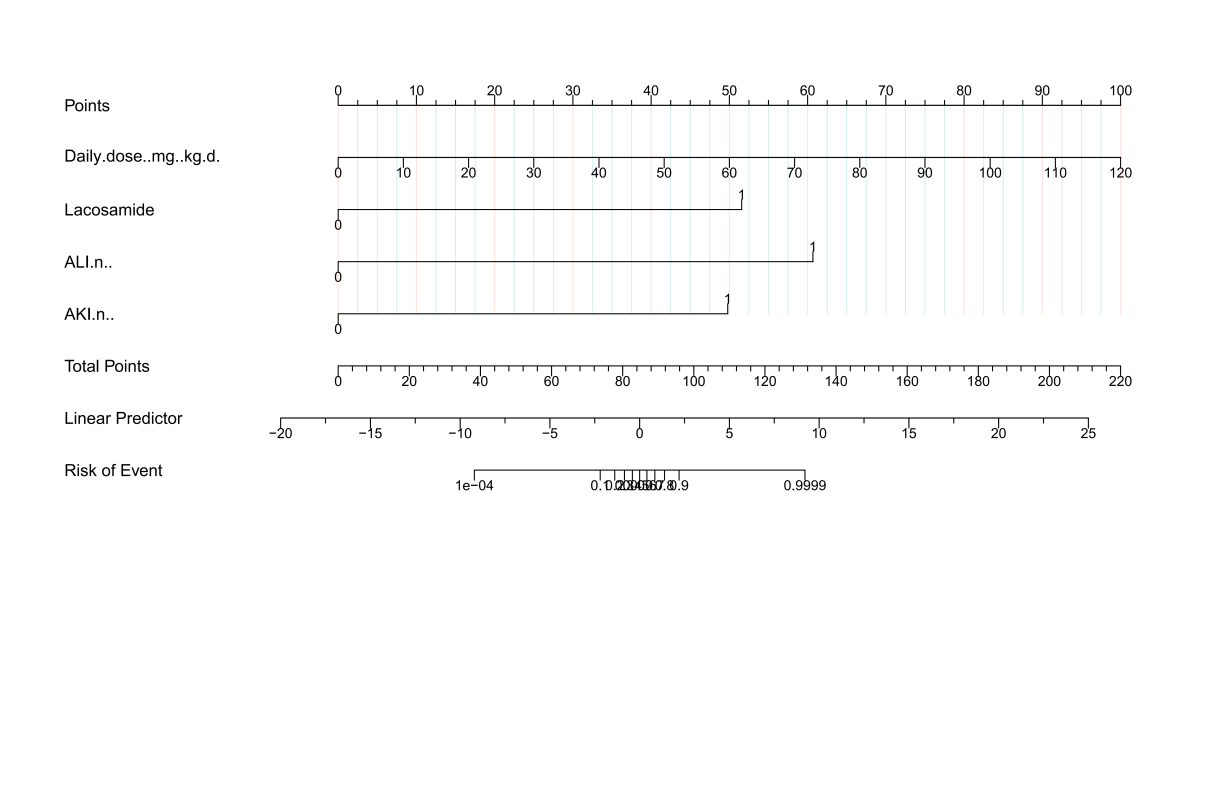


**Supplementary Figure 2** Nomogram to predict the probability of high blood concentration in Pediatric with Epilepsy.

**Supplementary Table 1.Multivariate regression analysis**

| **Variable** | **OR (95%CI)** | ***P* value** |
| --- | --- | --- |
| Daily dose(mg/kg/d) | 1.1 (1.05~1.16) | <0.001 |
| Lacosamide |  |  |
| NO | 1(Ref) |  |
| Yes | 6.42 (1.65~24.94) | 0.007 |
| ALI |  |  |
| NO | 1(Ref) |  |
| Yes | 53.33 (11.04~257.67) | 0.001 |
| AKI |  |  |
| NO | 1(Ref) |  |
| Yes | 46.29 (8.2~261.41) | <0.001 |

OR,odds ratios;ALI,acute liver injury; AKI,acute kidney injury


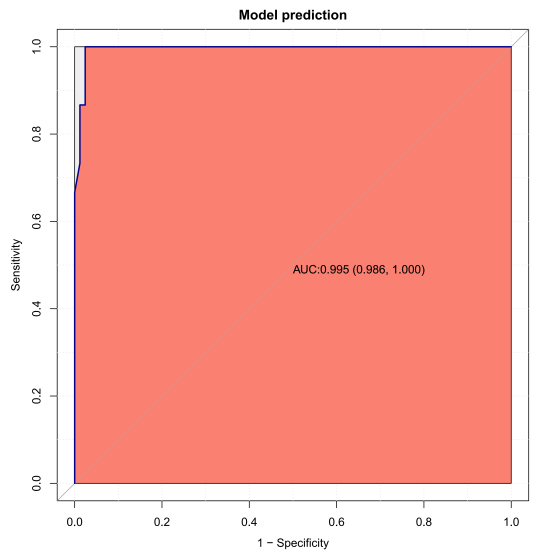

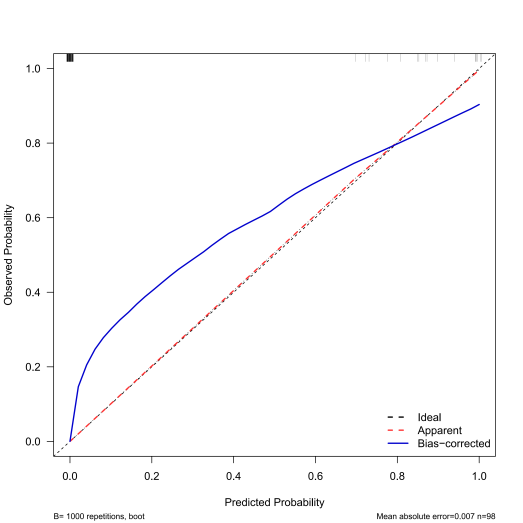


**
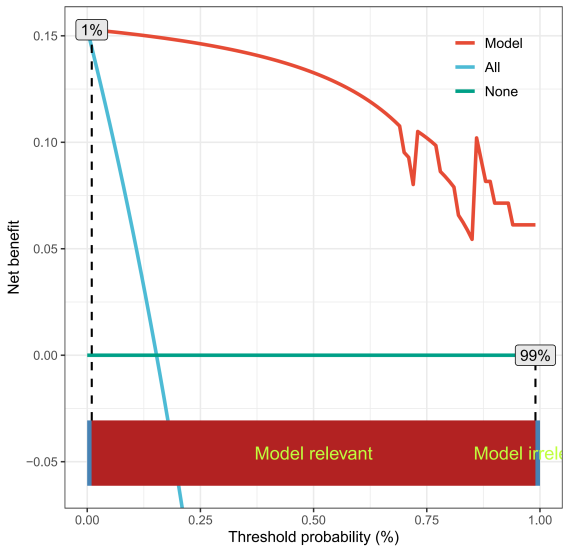
**

**Supplementary Figure 3** The discrimination and calibration assessment of the model

**2、Establishment and validation of the nomogram prediction model (Low vs. Therapeutic)**

Boruta algorithm identify the potential prognostic factors were weight,WBC, hemoglobin and meropenem (Supplementary Figure 4). Multivariate analysis further identified the variables were hemoglobin and meropenem (Supplementary Table 2).These factors were integrated into a multivariable logistic regression model, which was illustrated using a nomogram (Supplementary Figure 5). Similarly, the nomogram prediction model exhibits overfitting and unstable due to insufficient outcome events (n=23)(Supplementary Figure 6).


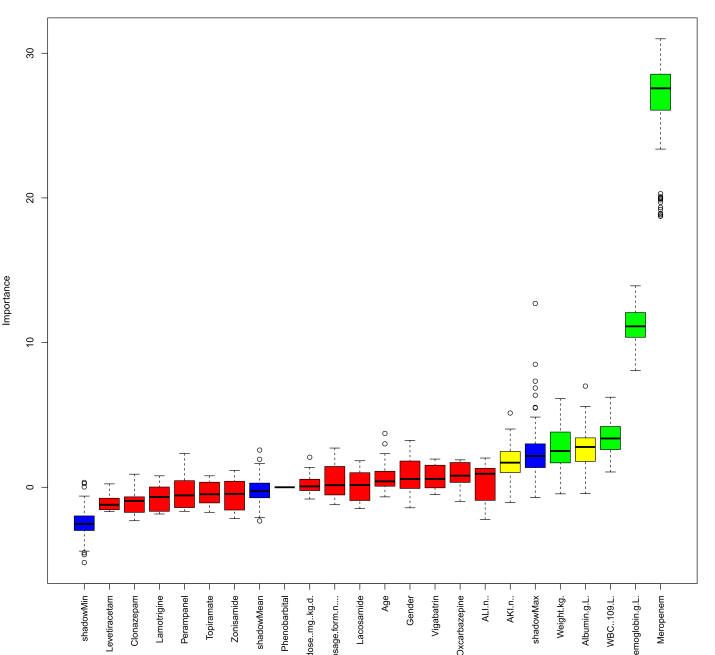


**Supplementary Figure 4** Feature selection based on the Boruta algorithm. The horizontal axis represents the name of each variable, while the vertical axis denotes the Z value of each variable. The box plot illustrates the Z value of each variable throughout the model computation. The green boxes denote significant variables, whereas the red boxes indicate insignificant variables.


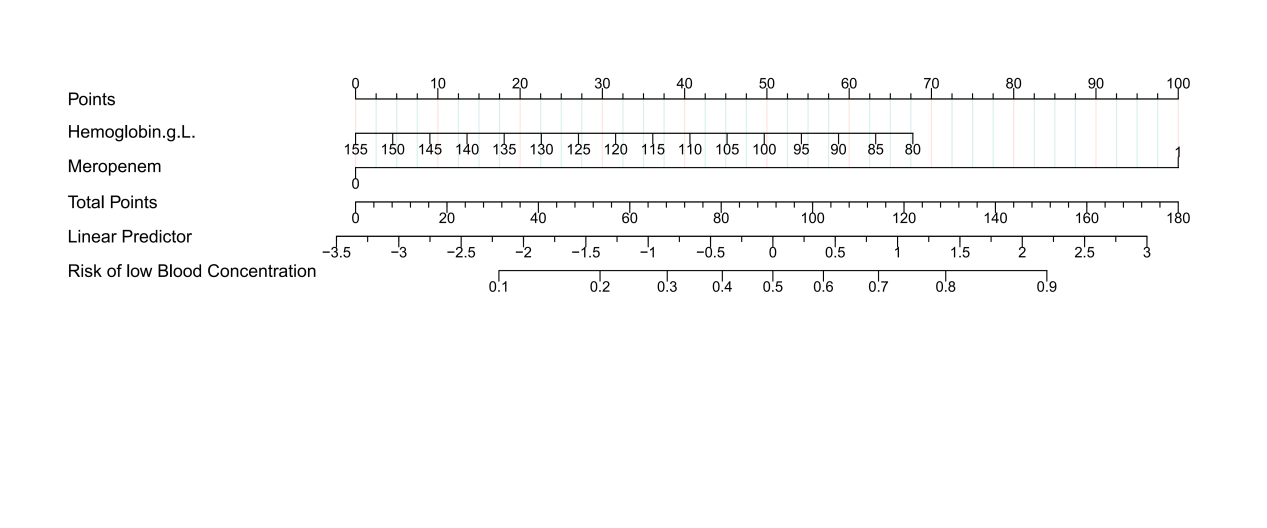


**Supplementary Figure 5** Nomogram to predict the probability of low blood concentration in Pediatric with Epilepsy.

**Supplementary Table 2.Multivariate regression analysis**

| **Variable** | **OR (95%CI)** | ***P* value** |
| --- | --- | --- |
| Hemoglobin(g/L) | 0.96 (0.93~1) | 0.028 |
| Meropenem | 41.48 (9.98~172.42) | <0.001 |


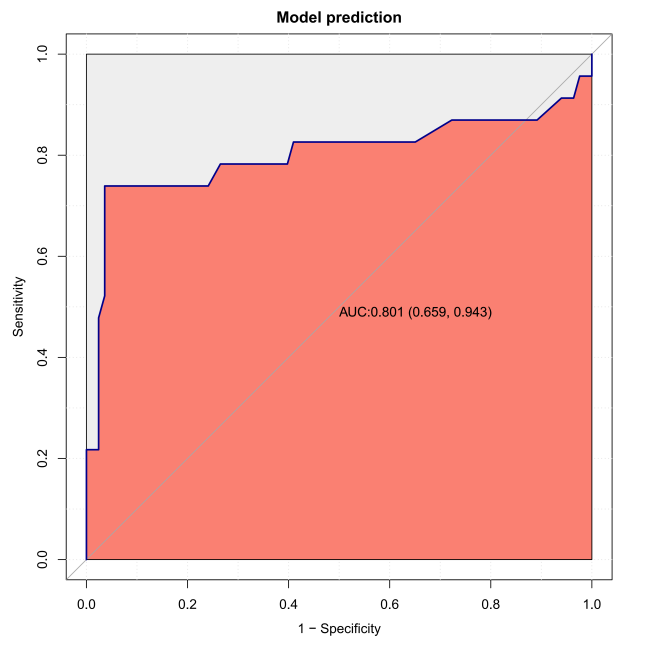

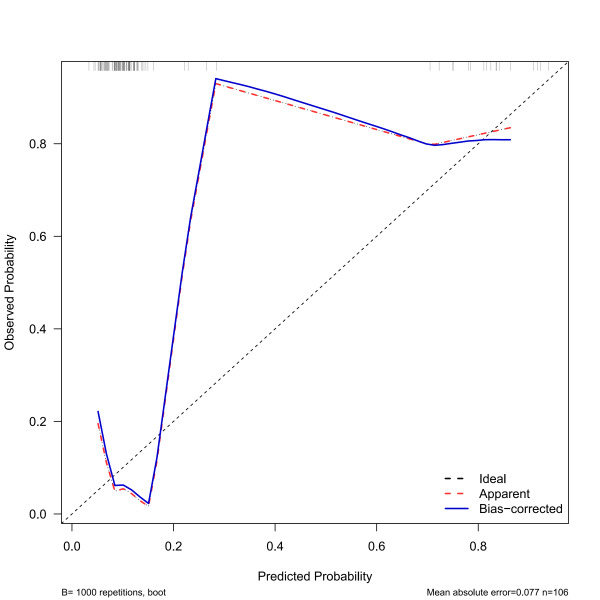


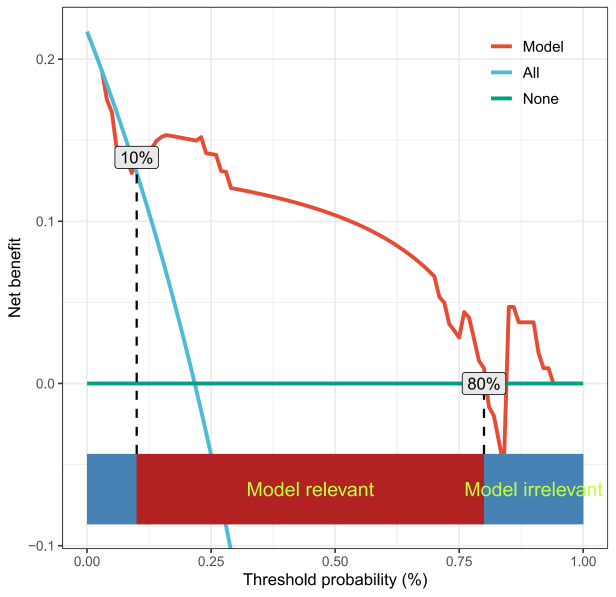

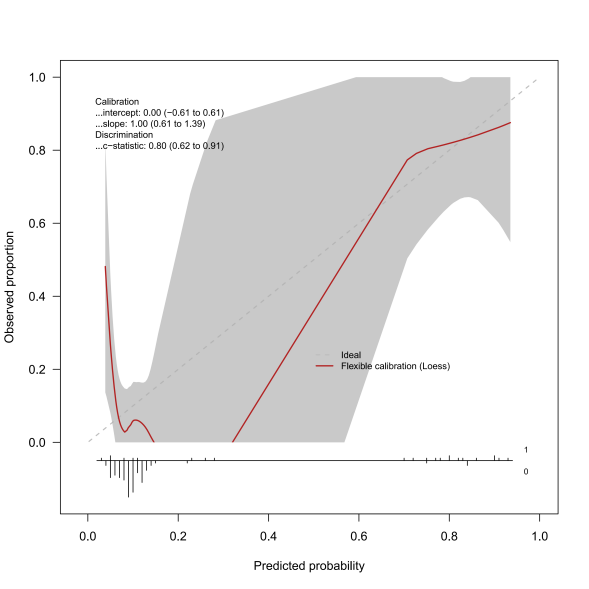


**Supplementary Figure 6** The discrimination and calibration assessment of the model

**3、Comparative analysis of effect sizes across three distinct groups**

We conducted separate multivariable analyses for low vs. optimal and high vs. optimal concentrations. We then systematically compared the effect directions of four predictors across the two subgroups. The results demonstrated that all variables exhibited identical effect directions across groups (OR>1), with no evidence of opposing effects(Supplementary Table 3). Meropenem showed an OR value of 1.90 (95%CI:0.18-19.64) in the high-concentration group. Although statistically insignificant due to limited sample size, its estimated direction aligned with that in the low-concentration group, further supporting directional homogeneity. Consequently, the suboptimal model did not mask any opposing effects but instead enhanced statistical power, enabling more robust estimation of overall nomogram effects.

**Supplementary Table 3.Comparative analysis of effect sizes across three groups**

| **Variable** | **Low-concentration**  **vs Therapeutic**  **OR (95%CI)** | ***p*** | **High-concentration**  **vs Therapeutic**  **OR (95%CI)** | ***p*** | **Suboptimal**  **vs Therapeutic**  **OR (95%CI)** | ***p*** |
| --- | --- | --- | --- | --- | --- | --- |
| Daily dose  (mg/kg/d) | 1.02 (0.97~1.07) | 0.495 | 1.1 (1.05~1.16) | <0.001 | 1.06 (1.02~1.1) | 0.006 |
| Meropenem | 41.48 (9.98~172.42) | <0.001 | 1.9 (0.18~19.64) | 0.588 | 17.39 (4.63~65.33) | <0.001 |
| AKI | 6.07 (0.95~38.83) | 0.057 | 46.29 (8.2~261.41) | <0.001 | 16.5 (3.44~79.18) | <0.001 |
| ALI | 1.21 (0.12~12.23) | 0.87 | 53.33(11.04~257.67) | <0.001 | 10.86 (2.82~41.87) | 0.001 |

**4、User Guide for the Valproate Nomogram**

***Purpose***

This nomogram predicts the probability of a suboptimal valproate concentration (trough level <50 μg/mL or >100 μg/mL) in pediatric patients with epilepsy. It is intended as a screening tool to identify high‑risk patients who may benefit from intensified therapeutic drug monitoring (TDM).

***How to use the nomogram – Step by step***

*Step 1* – Locate the patient’s values for each predictor

The nomogram includes four predictors: Daily dose (mg/kg/d),Acute kidney injury (AKI) : Yes or No,Acute liver injury (ALI) : Yes or No,Meropenem use : Yes or No.

*Step 2* – Read the points for each predictor

On the nomogram, each predictor has a “Points” scale at the top. Draw a vertical line from the patient’s value to the “Points” line to obtain the points for that predictor.

For dose: a continuous line; find the dose value and read its points.

For AKI, ALI, Meropenem: “Yes” gives a certain number of points; “No” gives 0 points.

*Step 3* – Sum the points

Add the points from all four predictors. This is the Total Points.

*Step 4* – Read the predicted risk

On the “Total Points” axis (bottom of the nomogram), locate the Total Points sum. Draw a vertical line downward to the “Risk of suboptimal concentration” axis. The value at the intersection is the predicted probability (as a percentage).

*Step 5* – Interpret the risk

Predicted risk ≥ 33.7% (the optimal cut‑off derived from our cohort) → classify as high risk. Recommend intensified TDM and careful clinical evaluation.

Predicted risk < 33.7% → low risk. Routine TDM is still advised, as occasional patients may have unmeasured risk factors.

***Example***

Patient:6‑year‑old child, weight 20 kg

Valproate dose: 25 mg/kg/d (i.e., 500 mg daily)

ALI: Yes, AKI: No, Meropenem: No

Steps: Dose 25 mg/kg/d → points ≈ 25 (from the dose‑points line)

ALI = Yes → points ≈ 40

AKI = No → points = 0

Meropenem = No → points = 0

Total points = 25 + 40 = 65

Total points = 65 → predicted risk ≈ 60% (from the risk axis)

Since 60% > 33.7%, this patient is high risk for suboptimal valproate concentration.

Clinical action: Initiate early and frequent TDM, and assess the direction of deviation based on individual factors (e.g., if ALI is present, the patient is more likely to have high concentrations; dose adjustment should be guided by serial TDM results).

***Important notes***

This tool is a screening aid,it does not replace therapeutic drug monitoring or individualised clinical decision‑making. Due to dosage alone is insufficient to reliably predict the risk of low concentrations,we adopted the minimum dose specified in the drug package insert(20mg/kg/d) as the applicable minimum dose condition for using this predictive model. The model measures total valproate concentration. In patients with hypoalbuminemia or renal impairment, free concentration may differ, the understanding of the nomogram should supplement with clinical judgment.
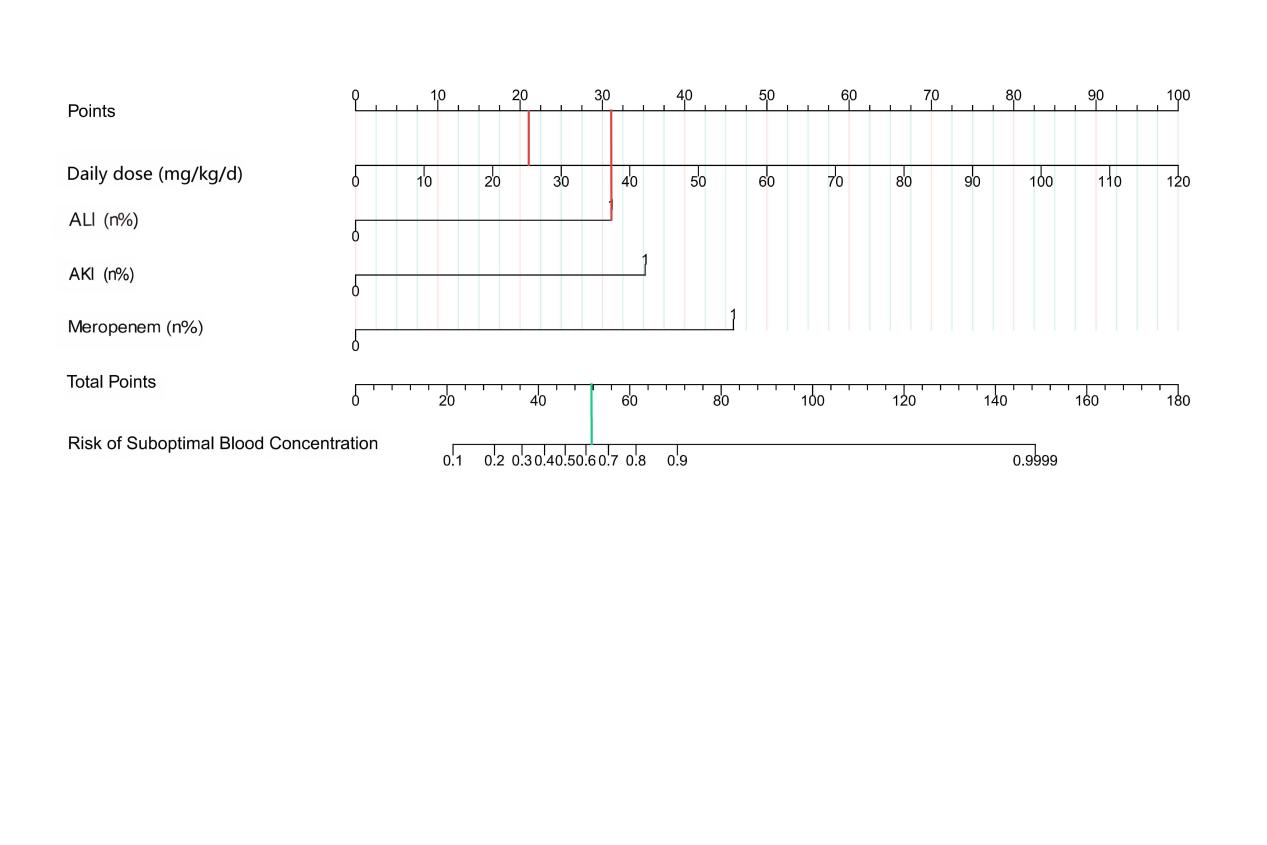


**Supplementary Figure 7** Nomogram for the User Guide

1. **Sensitivity analysis excluding the injection group**

To assess the influence of the injection group (n=2) on the study outcomes, we performed a multivariate logistic regression analysis once more after omitting these two patients (n=119). The results (Supplementary Table 4) indicated no significant alterations in the direction of OR values or P-values for core variables relative to the initial study. The data suggest that the principal conclusions of this study are not influenced by a limited number of patients in the injection group, hence illustrating the robustness of the results.

| **Model** | **Variable** | **Original analysis** | ***p*** | **Sensitivity Analysis** | ***p*** |
| --- | --- | --- | --- | --- | --- |
| **Low-concentration**  **vs Therapeutic**  **OR (95%CI)** | Daily dose  (mg/kg/d) | 1.02 (0.97~1.07) | 0.495 | 1.02 (0.97~1.07) | 0.495 |
|  | Meropenem | 41.48 (9.98~172.42) | <0.001 | 41.48 (9.98~172.42) | <0.001 |
|  | AKI | 6.07 (0.95~38.83) | 0.057 | 6.07 (0.95~38.83) | 0.057 |
|  | ALI | 1.21 (0.12~12.23) | 0.87 | 1.21 (0.12~12.23) | 0.87 |
| **High-concentration**  **vs Therapeutic**  **OR (95%CI)** | Daily dose  (mg/kg/d) | 1.1 (1.05~1.16) | <0.001 | 1.09 (1.03~1.16) | 0.002 |
|  | Meropenem | 1.9 (0.18~19.64) | 0.588 | 2.22 (0.21~23.14) | 0.504 |
|  | AKI | 46.29 (8.2~261.41) | <0.001 | 64.8 (10.78~389.49) | <0.001 |
|  | ALI | 53.33(11.04~257.67) | <0.001 | 88.89 (15.76~501.44) | <0.001 |
| **Suboptimal**  **vs Therapeutic**  **OR (95%CI)** | Daily dose  (mg/kg/d) | 1.06 (1.02~1.1) | 0.006 | 1.05 (1.01~1.09) | 0.015 |
|  | Meropenem | 17.39 (4.63~65.33) | <0.001 | 41.48 (9.98~172.42) | <0.001 |
|  | AKI | 16.5 (3.44~79.18) | <0.001 | 17.82 (3.7~85.82) | <0.001 |
|  | ALI | 10.86 (2.82~41.87) | 0.001 | 11.73 (3.03~45.41) | <0.001 |

**Supplementary Table 4. Sensitivity analysis excluding the injection group**
